# Facial expression recognition based on active region of interest using deep learning and parallelism

**DOI:** 10.7717/peerj-cs.894

**Published:** 2022-03-02

**Authors:** Mohammad Alamgir Hossain, Basem Assiri

**Affiliations:** Department of COMPUTER SCIENCE, College of Computer Science & Information Technology, Jazan University, Jazan, Kingdom of Saudi Arabia

**Keywords:** Optimized searching algorithm, Pose estimation, Rotation and correction, Parallelism, Deep learning, Active region of interest, Facial expression recognition, Convolution neural network

## Abstract

The automatic facial expression tracking method has become an emergent topic during the last few decades. It is a challenging problem that impacts many fields such as virtual reality, security surveillance, driver safety, homeland security, human-computer interaction, medical applications. A remarkable cost-efficiency can be achieved by considering some areas of a face. These areas are termed Active Regions of Interest (AROIs). This work proposes a facial expression recognition framework that investigates five types of facial expressions, namely neutral, happiness, fear, surprise, and disgust. Firstly, a pose estimation method is incorporated and to go along with an approach to rotate the face to achieve a normalized pose. Secondly, the whole face-image is segmented into four classes and eight regions. Thirdly, only four AROIs are identified from the segmented regions. The four AROIs are the nose-tip, right eye, left eye, and lips respectively. Fourthly, an info-image-data-mask database is maintained for classification and it is used to store records of images. This database is the mixture of all the images that are gained after introducing a ten-fold cross-validation technique using the Convolutional Neural Network. Correlations of variances and standard deviations are computed based on identified images. To minimize the required processing time in both training and testing the data set, a parallelism technique is introduced, in which each region of the AROIs is classified individually and all of them run in parallel. Fifthly, a decision-tree-level synthesis-based framework is proposed to coordinate the results of parallel classification, which helps to improve the recognition accuracy. Finally, experimentation on both independent and synthesis databases is voted for calculating the performance of the proposed technique. By incorporating the proposed synthesis method, we gain 94.499%, 95.439%, and 98.26% accuracy with the CK+ image sets and 92.463%, 93.318%, and 94.423% with the JAFFE image sets. The overall accuracy is 95.27% in recognition. We gain 2.8% higher accuracy by introducing a decision-level synthesis method. Moreover, with the incorporation of parallelism, processing time speeds up three times faster. This accuracy proves the robustness of the proposed scheme.

## Introduction

Facial expressions are an important way for humans to express emotions, and they play a noteworthy role in our day-to-day lives. Facial expression recognition (FER) is a multifaceted problem that has motivated people during the last few decades. The applications of FER are found in virtual reality, security surveillance (Hossain & Sanyal, 2016), driver safety, homeland security, human–computer interaction (HCI) (Jiwen, Gang & Jie, 2017), health care, medical applications, eyes’ disease diagnosis (Hossain, Samanta & Goutam, 2012), and many more. There are various types of applications based on face images in our daily life so FER has attracted many researchers to explore image processing. Research scholars propose various innovative approaches to recognize facial expression (Jun et al., 2018) and its authentication (Mohammad & Assiri, 2020) in the recent past. However, there is still lacking such a FER system enriched with a higher percentage of recognition accuracy and lesser execution time  (Hossain & Assiri, 2020a). There is the scope to incorporate new ideas and better techniques so that higher recognition accuracy can be achieved in a minimal time  (Daihong et al., 2021). There are challenges in FER because in recognizing any face, several factors are interrelated.

The researchers usually use normal digital images to recognize human faces for many reasons, *e.g.*, the cost of cameras, ease of understanding, and simplicity of implementation. This recognition scheme is divided into the following major phases:

• Processing of the facial image
• Training and testing image representation
• Classification of the desired expression

**Figure 1:**
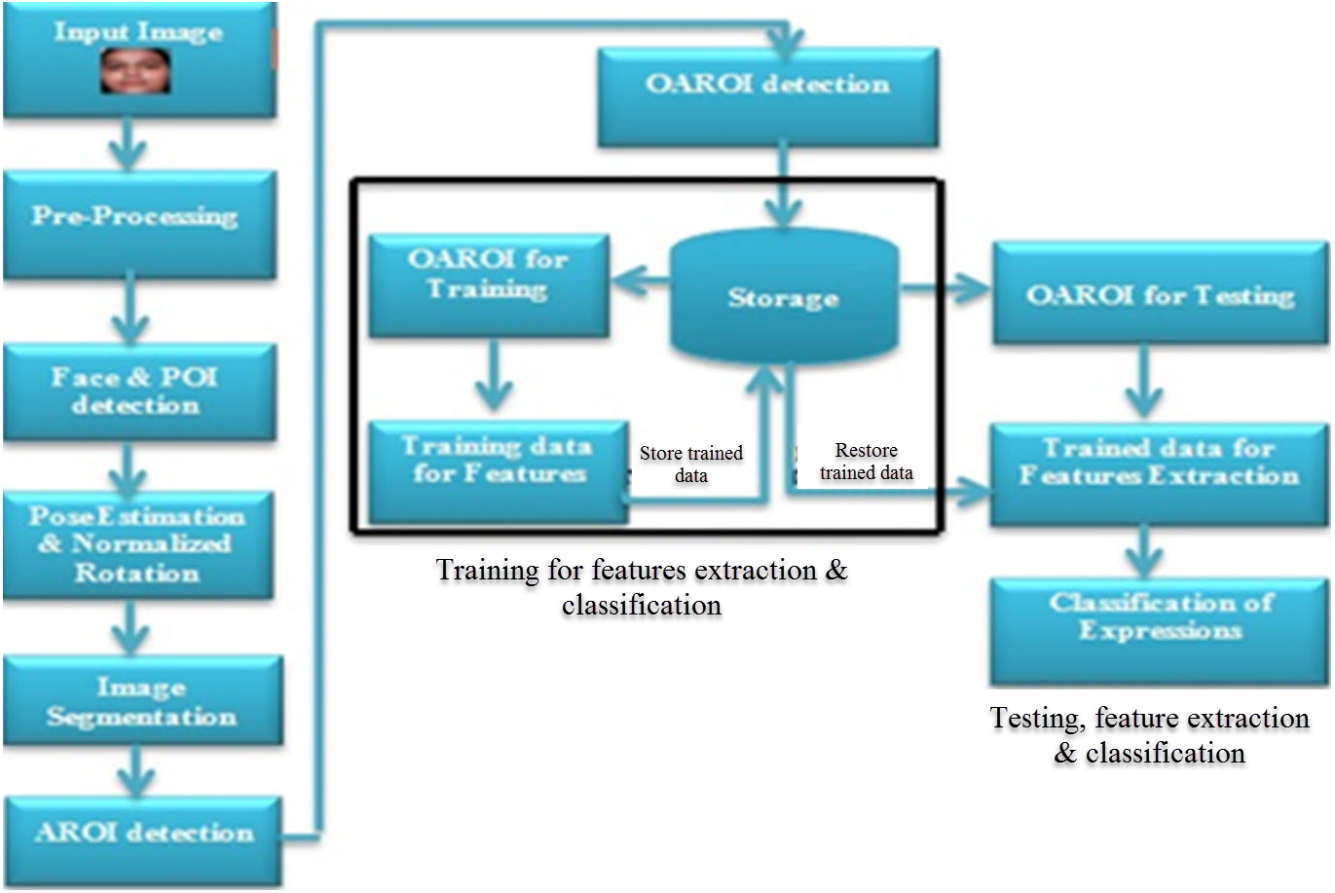
Proposed workflow diagram showing the three steps of expression recognition process.

**Processing of the facial image:** This part is vital to complete the whole process because it includes all the processes mentioned in the proposed workflow diagram (Fig. 1), namely: read an image, pre-processing, face image and point of interest (POI) (Daihong et al., 2021; An et al., 2019) detection, pose estimation (Hossain & Assiri, 2020b; Yongbin et al., 2019), image segmentation and AROI detection. A FER system is entirely dependent on the actual pixels of that face image. An optimized searching algorithm is incorporated to complete faster tracking. This algorithm is explained later and is called Algorithm-2.

**Training and testing image representation:** This part is concerned with preparing images for testing in the system. Here images are fed in for testing and training. Based on successful training, trained images are achieved and subsequently, the required features will be extracted. A ten-fold cross-validation technique is proposed for image set splitting for training and testing.

**Classification of the desired expression:** The prime goal of this paper remains in classifying the desired expressions out of five kinds (neutral, happiness, fear, surprise, and disgust). In this work, the Convolutional Neural Network (CNN) (Ran et al., 2019; Galea & Farrugia, 2018; Jae & Bumshik, 2020; Ferrari et al., 2018; Sun et al., 2018; Mollahosseini, Chan & Mahoor, 2016; Shen, Wang & Liu, 2013; Yahia, Mohammad & Hossam, 2020; AlBdairi, Zhu & Mohammed, 2020; Almeida et al., 2020; Boyina, 2020; Rajan et al., 2020) is incorporated to classify the expression from the trained data. To classify the desired expressions, features are extracted first from the trained image sets. We introduced CNN to classify three AROIs in parallel, so there will be three outputs. We made this classification because it is necessary for experimentation. We need to conduct experiments on both training and testing data sets. In this experiment, we proposed the decision-tree-level synthesis (Salmam, Madani & Kissi, 2016) method, which is illustrated in ‘Classification by decision-tree-level synthesis’.

This paper has a few prime contributions that are as follows.

1. The entire face is not used. Only four AROIs are chosen from the entire face to classify expressions.
2. A search technique called OAROI is proposed to search the similarity of the AROIs.
3. We propose a decision-tree-level synthesis-based framework to achieve improved accuracy in expression and its classification.
4. Parallel processing is applied to reduce execution time.

The remaining part of our manuscript is organized in the following way: Section 2 illustrates few “Related Works”. In Section 3, the “Proposed Methodology for Expression Recognition” depicts the designed frameworks for expression recognition. Sub-section 3.1 describes the “Data Processing”, Sub-section 3.2 illustrates “Rotation Using the Spatial Normalization Method”. Sub-section 3.3 explains the “Searching for the Optimized Active Region of Interest”. In Sub-section 3.4, “Parallel Face and Facial AROI Detection” is explained. In Sub-section 3.5, the “Classification by Decision-tree-level Synthesis” is introduced to achieve improved accuracy in expression classification. The experimentation and discussion are explained in Section 4. Lastly, we came to a conclusion that shows the robustness of our work. In this regard, we made the necessary comparison based on our experimental results and the results of other researchers. However, we have given the future direction of our work.

## Related Work

Human face tracking has become a promising area in the last few decades. To search for a few parts from an entire face for training is an emergent idea. Based on a few parts it is possible to gain all the required features. This unit overviews to extract features by incorporating appearance-based facial expression (Hossain & Sanyal, 2013) and emotion tracking techniques (Hossain, Zogan & Sanyal, 2018). To track facial expression accurately, it is necessary to use a better facial recognition technique. Due to the rising importance of accurate face recognition techniques scholars have investigated and proposed various techniques in the current time. Hossain & Assiri, (2020a) proposed a method for tracking and verification at minimal cost by using a pipeline computation method. They incorporated the famous Sinusoidal Head Model (SHM) and applied adaptive PDF (APDF) to estimate pose. The completion of the FER methods depends on active facial landmark points. A high-resolution camera is required to capture the images. The traditional emotion recognition schemes (Hossain, Zogan & Sanyal, 2018; Zhang et al., 2020) have some limitations because they follow self-measurement methods. Such schemes are not fit for quick surveillance. Self-reporting systems are incapable of classifying emotion quickly. Since emotion is associated with physical appearance, some investigators (Hossain, Zogan & Sanyal, 2018; Hossain, Samanta & Sanyal, 2012a) have used the physical signs for emotion recognition. A few proposed models track people based on the lips (Hossain & Sanyal, 2014), mouth, and eyebrows. There are some methods proposed by researchers to detect facial expressions by using various methods. Authors have suggested the FACS (facial action coding system) (Clark et al., 2020) to identify facial action. They presented face motions by using the Action Units (AUs) from the facial landmark-point. Correct recognition of AUs is essential when it is measured employing FACS, although it is hard to identify all the types of AUs. The scholars (Clark et al., 2020; Hossain & Goutam, 2012) have classified FER by applying FACS, geometric algorithm, and region-based systems  (Hossain & Goutam, 2012) and as well as appearance-based approaches to extract features  (Happy & Routray, 2015; Lajevardi & Hussain, 2021). In a geometric-based system (Lajevardi & Hussain, 2021), the locations or regions of a face (like eyes, eyebrows, mouth, and lips) are extracted from features-vector that identifies facial geometry. A geometry-based approach should attain alike performance to that of an appearance-based scheme. Appearance-based approaches (Hossain & Goutam, 2012; Happy & Routray, 2015; Lajevardi & Hussain, 2021) use all features related to facial appearance. A few investigators (Happy & Routray, 2015; Lajevardi & Hussain, 2021) have separated facial regions into a few blocks so that feature extractions become easier. Improved accuracy is attained using these techniques (Lajevardi & Hussain, 2021). It has been shown that facial features are directly related to expressions. These expressions have a direct correlation with the mouth, eyes, lips, nose, *etc*. Their poses, positions, and shapes are required for classifying facial expressions. These works are inspiring. These lead us to find out the concept of active regions of interest (AROI) on a human face. Zhang & Wang (2020) proposed face recognition algorithms for fatigue detection by using video image information.

A few researchers (Hossain & Sanyal, 2012a; Alamgir & Sanyal, 2012d; Hossain, Samanta & Sanyal, 2012c) recognize expressions like smiles, panic, and other expressions. They incorporated a haar-like method in detecting face and used the AdaBoost method to select features. This method had also been incorporated into the Open Source Computer Vision (OpenCV) Library (Fan et al., 2012). An OpenCV technique uses API tool to identify activity from a desktop computer in a real-time application. So the research scholars have used OpenCV to recognize facial image. Face alignment is an essential scheme to achieve better performance in FER. The objective of an alignment is restricted to a predefined AROI (Sun et al., 2018) from a face image. Scholars (Liu et al., 2016) proposed how to identify facial landmarks based on shape indexed features. A few companies, like Microsoft, Face++ have applied their state-of-the-art techniques to identify facial landmarks. They used their API tools and techniques in identifying face landmark points.

In general, it is found in FER schemes that the whole area of a face is taken into account. But if the whole face is divided into few regions then there must have few benefits. In the article by Liu et al. (2016) it was shown that a whole face-image is separated into 6×7 identical patches. Then, the LBP (Local Binary Patterns) features are pulled out from these patches. The SVM (Support Vector Machine) classifier is applied to classify facial expressions. There are some drawbacks to this method in fixing regions’ sizes and their positions. Scholars proposed the AdaBoost (Geng et al., 2019) method to get rid of this problem and they used LBP features in their work. They achieved two features AdaBoost and LBP. They used sub-window in their application and scaled the face as per the window size. Liu et al. (2016) separated the face into 8x8 and suggested multi-task sparse (MTS) technique to find the active areas. They pinpointed active areas within the nose, mouth, eyes, and lip regions. We have incorporated AROIs in this paper and proposed a searching algorithm called OAROI. Hossain et al. propose the Gabor-wavelet (Hossain & Sanyal, 2012b) and genetic algorithm (GA) (Hossain, Samanta & Sanyal, 2012b) for feature extraction and classification. They showed that these techniques are efficient than that of a geometric-based method. These kinds of features are expensive to extract in terms of computation. However, the proposed LBP as a low-cost scheme to extract dissimilar features from a facial expression. LBP is broadly used to identify facial expressions  (Alam & Jianu, 2017) due to its performance.

During the last couple of years, it is noticed that mutation technique  (Hossain & Sanyal, 2012b) and GA is applied in FER. Liu et al. (2016) addressed Boosted Deep Belief Network (BDBN) to identify face. The BDBN had a small number of weak classifiers and for each of them is applied for an expression. They conducted their experiment on both CK+ (Mollahosseini, Chan & Mahoor, 2016; Shen, Wang & Liu, 2013) and JAFFE (Mollahosseini, Chan & Mahoor, 2016) databases and attained 96.7% and 91.8% accuracy in recognition. This accuracy is very high as compared to other methods but the BDBN needs more time to train its data sets. The expected time required for training data was almost 8 days. Ding, Zhou & Chellappa (2017) proposed the FaceNet2ExpNet method and they designed a two-folded scheme. Initially, the face-net is applied to systematize FER at the pre-training stage. However, they completed the testing of the data set in a connected layer. Zeng et al. (2018) proposed FER classification by using DSAE (deep sparse auto encode). They used three categories of classifiers namely LBP, gray value, and HOG.

In recent times Manda, Bhaskare & Muthuganapathy (2021) used the deep learning (Zeng et al., 2018; Rajeev, Vishal & Rama, 2019; Guosheng et al., 2018; Nada & Heyam, 2020) method to classify engineering models by using CNN (Lopes et al., 2017). They used huge datasets in their experiment and applied GPUs technique to minimize the computation time. It is addressed in Geng et al. (2019) that if ANN (artificial neural network) (Suri, Vatsa & Singh, 2020) is applied with CNN (Lucey et al., 2010; Mollahosseini, Chan & Mahoor, 2016; Yahia, Mohammad & Hossam, 2020; Almeida et al., 2020; Rajan et al., 2020) for detecting face then more accuracy can be gained. These detection methods are associated with correctness and recall rates, so they can be evaluated and applied in object detection. It is found that among the experiment results that the best one is the ANN method in terms of accuracy. Apart from the aforementioned algorithms, there are a few approaches like CNN to identify people by face recognition where the influences of poses, viewpoints, and illuminations have direct effects by tracking. The CNN is one kind of deep learning technique (Manda, Bhaskare & Muthuganapathy, 2021; Samadi, Akbarizadeh & Kaabi, 2019; Carletti et al., 2020; Shiqing et al., 2019; Rajeev, Vishal & Rama, 2019; Shen, Wang & Liu, 2013; Boyina, 2020) that can obtain features from used training data. It achieves features with a combination of convolutional and its sub-layers. This is surveyed by using a set of fully connected layers. In recent years, CNN is widely being used because of its proficient performance (Yahia, Mohammad & Hossam, 2020; Rajan et al., 2020). With the incorporation of this technique better performance is achieved and they have shown it in performance matrix (Sun et al., 2018; Yahia, Mohammad & Hossam, 2020) form. A few studies had been completed based on deep learning using CNN (Manda, Bhaskare & Muthuganapathy, 2021; Carletti et al., 2020; Shiqing et al., 2019; Sun et al., 2018; AlBdairi, Zhu & Mohammed, 2020; Saranya et al., 2020) and Machine Vision (MV) because MV is an appropriate method for achieving a high-performance rate. It plays a significant role together with Artificial Intelligence (AI) and Machine Intelligence (MI). Many scholars showed their investigational results based on deep learning (Jin, Cruz & Gonçalves, 2020; Jiwen, Junlin & Yap-Peng, 2017; Rajeev, Vishal & Rama, 2019; AlBdairi, Zhu & Mohammed, 2020; Saranya et al., 2020) and MV (Moghaddam, Akbarizadeh & Kaabi, 2019; Tirandaz & Akbarizadeh, 2016; Hossain, Samanta & Sanyal, 2013) techniques. Lopes et al. (2017) suggested a new approach to recognize facial expression by merging CNN with pre-processing like sample generation, correction of rotation, normalization of intensity, and so on. In this process, they attained 96.76% accuracy on the CK+ database with reduced time. The proposed method deals with various things so that it will be (i) faster in terms of access and process, (ii) have better performance in recognition. The proposed CNN achieves high accuracy in recognition with minimal training time. Apart from that, AROI is proposed to classify and identify the active regions for expected expressions as an alternative for the whole face region. Happy & Routray (2015) designated 19 active landmarks over the face. They extracted LBP features from nineteen active landmarks but only four landmarks from top were studied for classification. AlBdairi, Zhu & Mohammed (2020) have proposed that their CNN-based deep learning method has achieved 96.9% accuracy in the verification of the human face. Saranya et al. (2020) have proposed that their proposed CNN- and LSTM-based method has achieved facial expression recognition with 81.60%, 56.68%, and 95.21% accuracy by using MMI, SFEW, and their data set respectively.

Liu et al. (2014) and Moghaddam, Akbarizadeh & Kaabi (2019) have tried to speed up the processing time by applying various techniques (Almeida et al., 2020) in their research, but few of them became successful. Here processing time is reduced to a greater extent, although the measurement is done in floating-point, not in the exact value. Geng et al. (2019) used parallelism in their experiments. During application, they applied both parallelism and pipeline in their application.

## Proposed Methodology for Expression Recognition

The proposed FER methodology is explained in sub-section 3.1. And it is elaborated through sub-methods. However, the other sub-sections are entitled “Rotation using the Spatial Normalization Method” (sub-section 3.2), “OAROI searching” (sub-section 3.3), “Parallel and facial AROI detection” (sub-section 3.4), and “Classification by the decision-tree-level synthesis”(sub-section 3.5).

### Proposed methodology in general

In completing data processing, the following three phases are needed to work together. The three phases are (a) pre-processing of data; (b) training of classified data, and (c) testing of classified data. Figure 1 shows the flow diagram and illustrates the details of all the used steps. During the recognition process, these are followed. Phase-1 begins by accepting an input image into the system and it ends by detecting AROI. In phase-2, OAROI is detected first from the identified AROIs and then the training data sets are prepared for completing the testing in phase-3. Three operations are carried out in phase-3. The operations are (i) testing based on the OAROI data set, (ii) completion of the feature extraction training data being trained, and finally, (iii) classification of FER is completed.

#### a. Pre-processing of data

In pre-processing of data, images are being fed as input into the system and the required filtering techniques are applied. We applied weighted vector directional filter (WVDF) (Hossain, Samanta & Sanyal, 2012c) and SPAM filter (Shiqing et al., 2019). The mentioned filters are applied to minimize noises from the image. Moreover, pose estimation and rotation of an image for normalization are done as per requirement. To detect accurate facial expressions, pose estimation is an important aspect. Thus, the famous Adaptive Sinusoidal Head Model (ASHM) (Mohammad & Assiri, 2020) is applied, and it will be explained in detail in ‘Rotation using the Spatial Normalization Method’.

**Figure 2:**
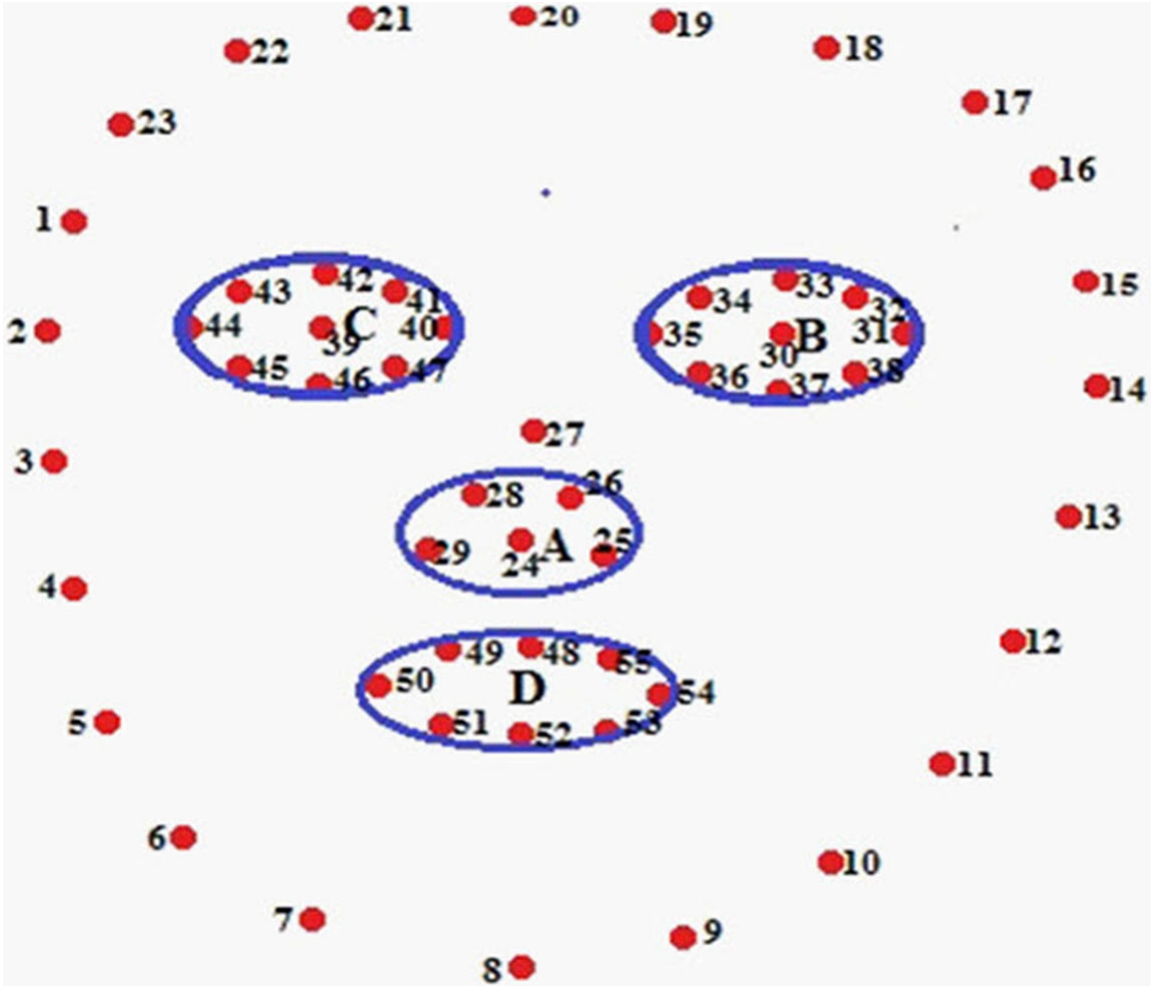
Finding POI and AROI. Nose-tip (A), LE region (B), RE region (C), Lip region (D)

Now, it is necessary to detect landmarks points in the face image. These landmark points are known as POIs and Regions of Interest (ROIs) as shown in Fig. 2. Figure 2 depicts the AROIs. Four AROIs are taken to experiment out results. We gained the maximum accuracy in recognition from these four AROIs.

**Figure 3:**
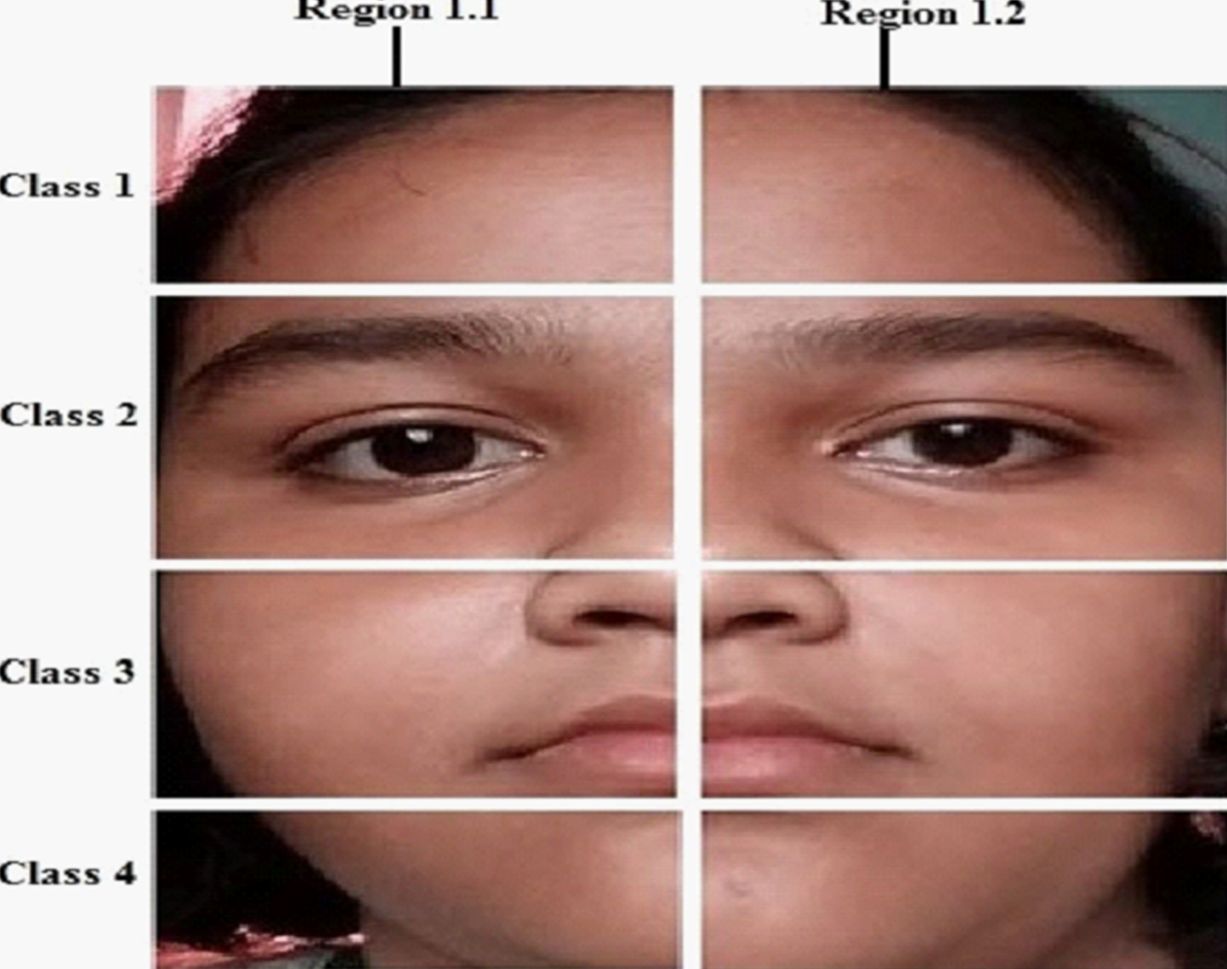
Face image is divided into four classes and eight regions.

Then a segmentation method is applied to divide a face image into 4 classes and 8 regions as shown in Fig. 3. The classes are the forehead, eyes, nose, and mouth areas, where every class is divided vertically into two halves.

The face image is segmenting an image into four classes and eight regions. We have chosen AROI from those segmented regions. These AROIs are nose-tip (A), left eye (B), right eye (C), and lip (D) as shown in Fig. 2.

#### b. Training of classified data

Now, an optimized searching algorithm is applied called OAROI. This scheme is illustrated in ‘Optimized Active Region of Interest (OAROI) searching’. To prepare data for training, as stated in the second phase of the proposed system, the following methods are incorporated.

a. An algorithm is applied, Algorithm 2, that will detect the OAROI and it will measure how much area will be considered for the most optimized area with the best result.
b. The data set is prepared for training data.
c. The training data set is ready and stored in the pre-defined memory storage location.

#### c. Testing of classified data

The testing data set is classified and segregated to prepare the trained data set. In this way, the classification is completed. However, the AROI-based CNN method is incorporated to gain the features for classification. The three regions left eye, right eye, and lip are considered. These three AROIs are trained and classified in parallel using CNN (explained in 3.4). The final classification is accomplished based on decision level synthesis method (explained in Classification by decision-tree-level synthesis’).

### Rotation using the spatial normalization method

It is found that a few images are askew due the motion of the camera. This would have affected the accuracy of recognition. To get rid of this situation and improve the approximation, we apply a head-pose estimation algorithm, called ASHM (Hossain & Assiri, 2020a). It has been shown (Hossain & Assiri, 2020a) that with ASHM, better results are achieved. We applied an adaptive sinusoidal head model (ASHM) to recognize and correct the head pose. We also applied PDF (Probability Density Function) along with ASHM. The Pose Matrix Pm was constructed first from the frame. The pose vector has a Rotation Matrix (Rm) and Transformations Matrix (Tm) for each frame, f. Each pose vector has an Rm and a Tm. *i.e.*,

(1) \begin{eqnarray*}{P}_{m}= \left[ \begin{array}{@{}ll@{}} \displaystyle {R}_{m}&\displaystyle {T}_{m}\\ \displaystyle {{T}_{m}}^{T}&\displaystyle 1 \end{array} \right] = \left[ {P}_{{m}_{1}} \right] \left[ {P}_{{m}_{2}} \right] \left[ {P}_{{m}_{3}} \right] \left[ {P}_{{m}_{4}} \right] \end{eqnarray*}

where Rm ∈ *Rm*_(8×8)_ and Tm ∈*Rm*_(8×1)_ are vectors after transformations by Tm. Pm_1_, Pm_2_, Pm_3_, and Pm_4_ are pose vectors.

The ASHM is applied to all frames starting from frame 1. In this way, by accessing all the frames, accurate features are attained from the trained images. We applied the LKT (Lucas Kanade Tracker) algorithm in the process of features extraction. These features have a straight association to the segmented classes. The identified AROIs are directly associated with head motion. However, it is necessary to apply the appropriate projection technique depending on the model of the camera. So a connotation can be established between a 3-D and a 2-D image plane.

**Figure 4:**
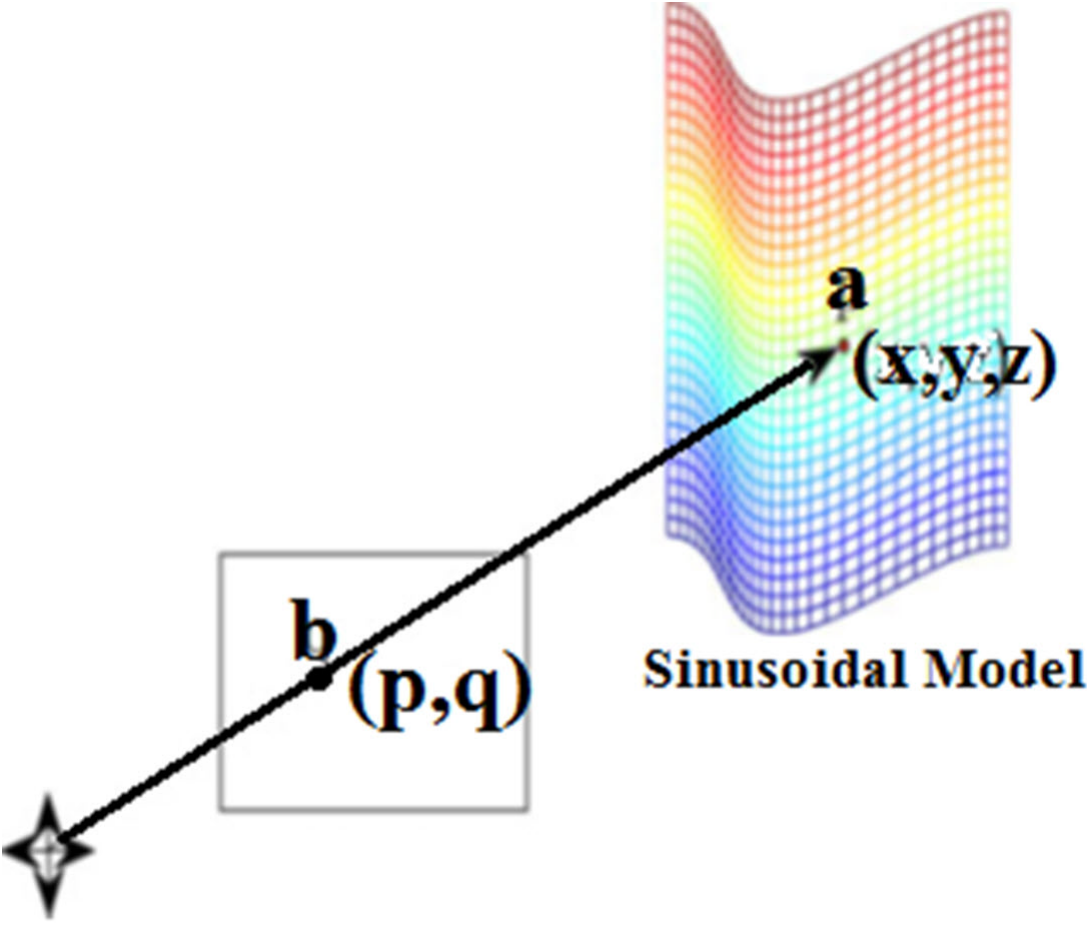
A 3-D point b onto the image plane.

Figure 4 illustrates an association among the 3-D points (indicated by *a* = (*x*, *y*, *z*)^*T*_*m*_^ on the sinusoidal area) and its prediction point (indicated by *b* = (*p*, *q*)^*T*_*m*_^) on the image plane. Here (p & q) are evaluated following the Eqs. (2) and (3).

(2) \begin{eqnarray*}p=d \frac{x}{z} \end{eqnarray*}

(3) \begin{eqnarray*}q=d \frac{y}{z} .\end{eqnarray*}

where d is the foretold distance from camera. Meanwhile 2-D features ensure different inferences in constructing pose-related information, two factors have a direct relation to feature extraction:

1. Intensity of features.

2. Applied tracking method in 3-D form.

Moreover, to overcome this situation, a rotation correction algorithm is incorporated. The positions of that part of AROIs maybe eyes, maybe lips, or both.

**Figure 5:**
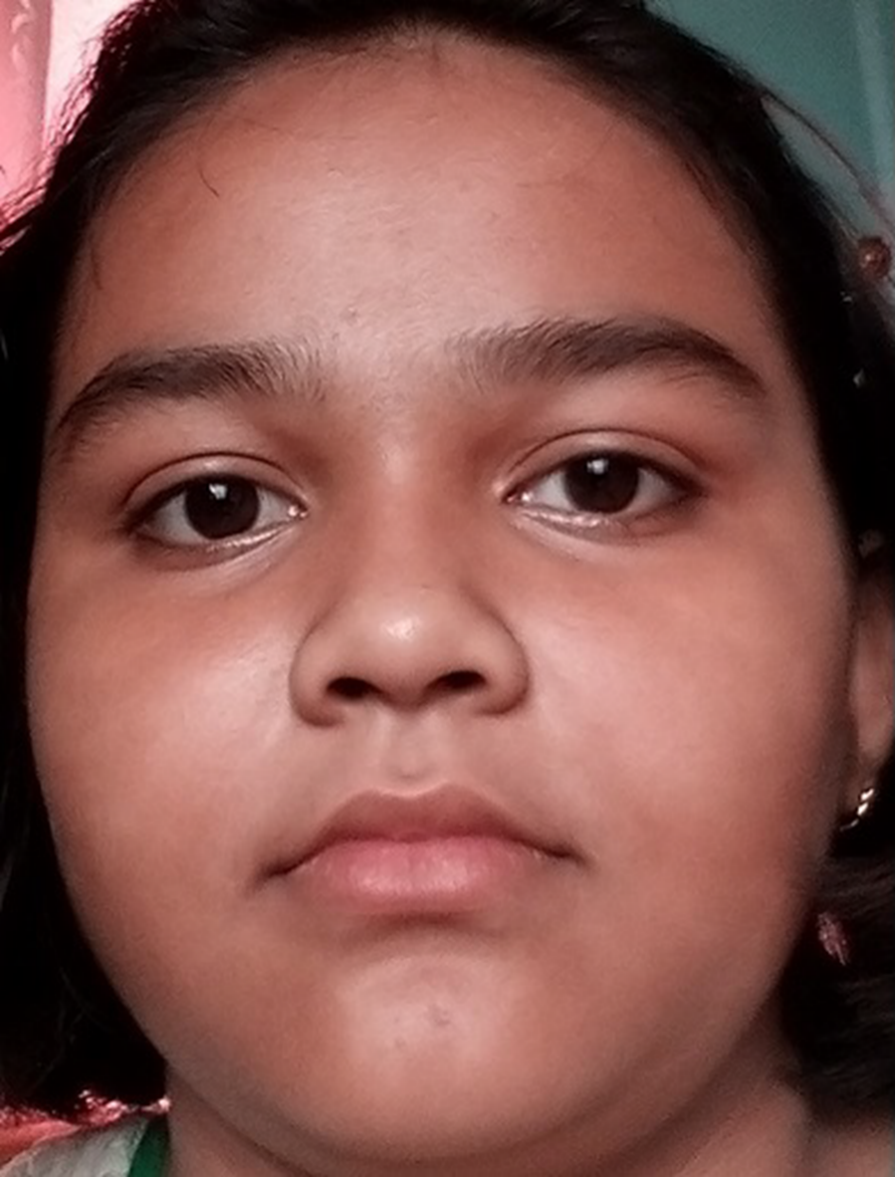
Normal image.

**Figure 6:**
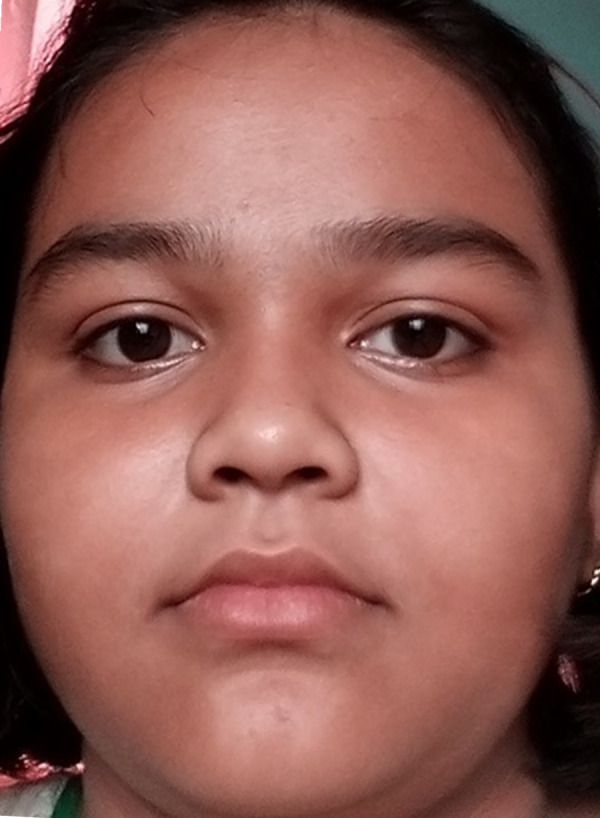
Image after rotation.

**Figure 7:**
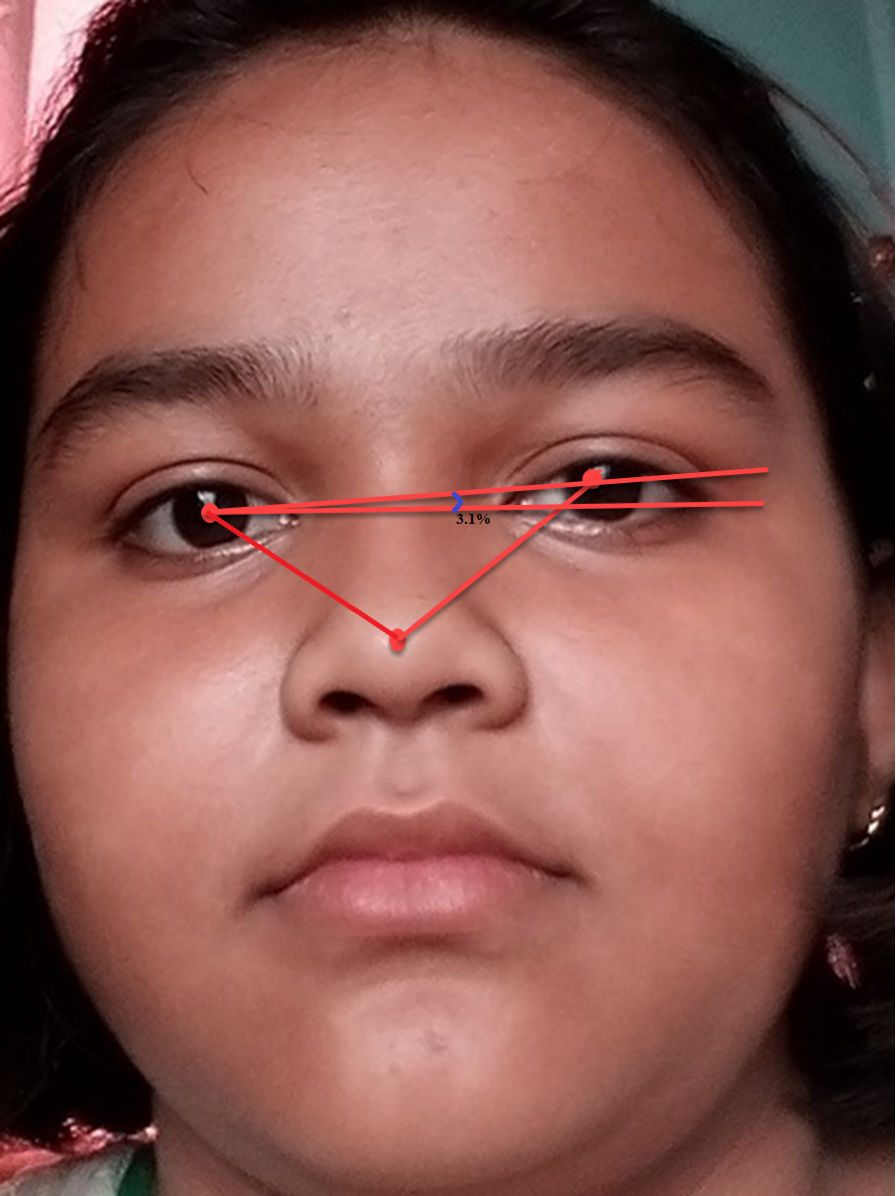
3.1% of rotation required to get the proper head pose.

**Figure 8:**
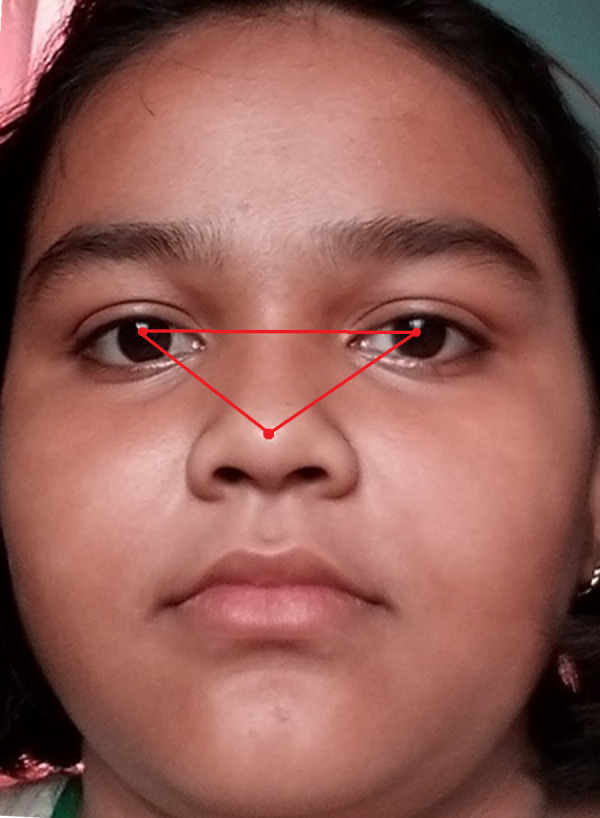
Image after rotation & normalization.

Figure 5 shows the original image without any alteration. Figure 6 shows the image after rotation of 3.1% required for normalization. Figure 7 shows the connection between two eye-lids. They are associated by a red line over the horizontal axis. It is used to achieve the adjusted pose. Figure 8 shows the image after rotation & normalization. Indeed, the angle in the middle of the two lines must be corrected. We applied the rotation and normalization method to gain the proper pose. In this process, 3.1% rotation is required to connect two eyelids. It is as shown through Figs. 5–8 respectively. The process of rotation has been demonstrated in the following paragraph.

Let (x1, y1) and (x2, y2) be left-eye and right-eye centers. Now let us calculate the angle *θ* by applying the equation no 1. So the image needs to be rotated to the extent of *θ*.

(4) \begin{eqnarray*}\theta =\tan \nolimits \left( \frac{{y}_{2}-{y}_{1}}{{x}_{2}-{x}_{1}} \right) .\end{eqnarray*}

Equation (4) is for rotation by the angle *θ*. The face-image size may vary after rotation. In this situation, a difficulty arises in classifying FER with increased accuracy. During rotation, if there exists a huge background part of the face area then a problem may arise. Although, the background part has less significance in recognizing facial expressions. As a consequence, we used the Spatial Normalization Method (SNM) to normalize the AROI areas of the face. The SNM is carried out based on a central point (the nose tip) of the face image. Let the length of the facial AROI be ∞ and the width is *β*. In this literature, it is set that ∞ = *β* = 120 pixels. Figure 6 shows the association between both left eyes of the face image and rotation is applied as required. After the SNM, facial POIs and the AROIs are spotted once more.

**Figure 9:**
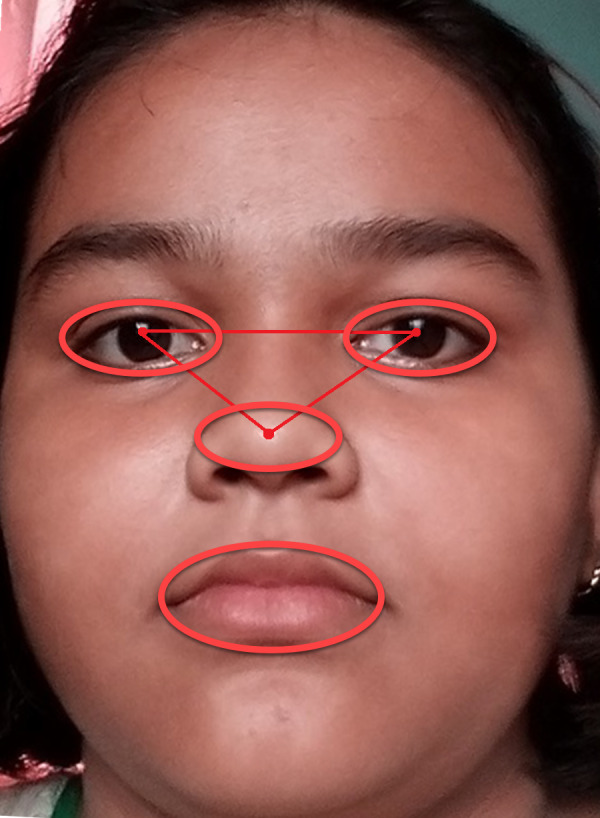
Examples of active regions with two scales, surrounded by red ellipses.

Figure 9 explains how the AROIs are being scaled. Here ellipse shape is considered. We considered geometric shape because it becomes easy to scale all the required parts of a human face. In this manuscript, three active regions (AROIs) have been taken for our experiment.

### Optimized active region of interest (OAROI) searching

The AROIs are detected based on four classes of segregation. The researchers (Hossain & Assiri, 2020a; Clark et al., 2020; Lopes et al., 2017) proposed that few regions are not active at all. Scholars (Lopes et al., 2017) separated a face area into 8*8 patches. They select active regions from 64 patches. These active patches are found within the eyes, nose, and mouth regions. It is a patch-based method only. The identified AROIs are shown in Fig. 9. The strong features are found in the AROIs. The strong features is considered by its feature weight because they yield more trustworthy information. The feature weights are extracted from nose tip, left eye, right eye, and lip regions and they are named FWA, FWB, FWC, and FWD respectively. The achieved feature-weights are as follows:

• Nose-tip FWA (24, 25, 26, 28, 29) = 0.0286943
• Left Eye FWB (30, 31, 32, 33, 34, 35, 36, 37) = 0.0129583
• Right Eye FWC(39,40,41,42,43,44,45,46,47) = 0.0173181
• Lip FWD(48,49,50,51,52,53,54,55) = 0.0319725

In the experiment, similarity amongst the similar AROIs of dissimilar expressions differs based on their sizes. During the choice of AROI, an optimized technique is applied so that those active AROIs should be chosen where maximum similarity is matched with the sizes of the regions. A searching method is applied so that the Optimized AROIs are found directly. In doing this, the hash distance (Lopes et al., 2017) algorithm is applied. There is a well-established rule concerning hash distance. There is a thumb rule that if the hash distance is smaller then similarity should be greater. The algorithm-1 is used to estimate the hash distance between two images.

Algorithm 1: Measurement of Hash Distance

Input: 2 images

Output: Measured Hash Distance between 2 images

a. Find AROIs (Nose-tip, Left-eye, Right-eye and Lip regions)
b. Choose Central-point (Nose-tip)
c. Resize images to 120*80
d. Convert image from RGB to gray.
e. Compute Mean value (MV) from gray image
f. Set POI = 1; if MV > = average, otherwise POI = 0.
g. Output will be the Hamming Distance based on a new gray value (0 or 1).

The following symbols are introduced to illustrate the optimized searching method. Let I = {1, 2, 3, 4,…,7} be the no of subjects in (CK + & JAFFE) image data sets. Here [J = {1, 2, 3, 4, 5}] are five expressions. (*i.e.*, 1 if for Normal; 2 is for Happy; 3 is for Fear; 4 is for Surprise; and 5 is for Disgust). Set Eij, i ∈ I, j ∈ J. This implies that the ith subject with jth expression.

**Figure 10:**
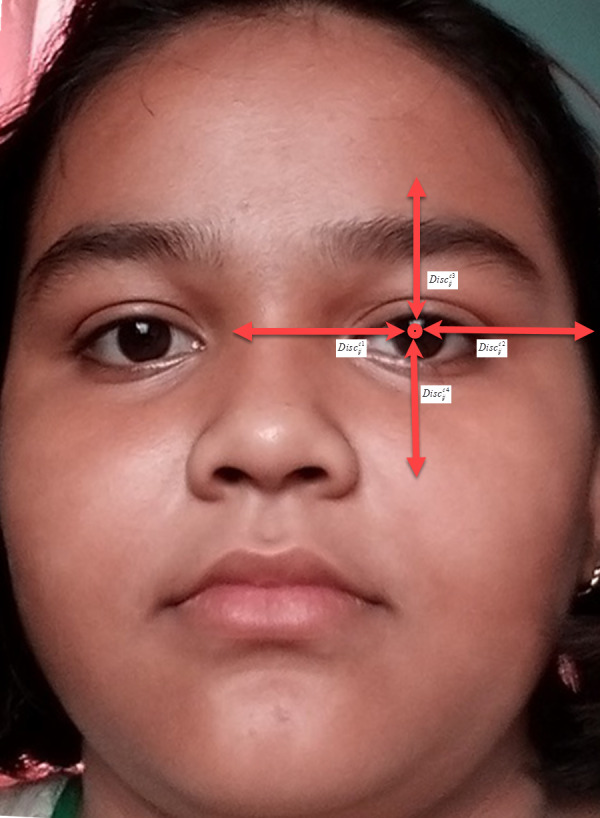
The image shows how to measure the angle of rotation required mathematically.

Here [C = {Left-eye, Right-eye, Lip regions}] denotes the classes of AROIs. Lc is equal to {1,2, …, bound_*c*_} where c ∈ C which is taken as radius of AROIs. Its size is c. After measuring size we estimate the boundary as named bound_*c*_. There are four directions *D* = {1, 2, 3, 4} identified as 1 is on left, 2 is on right, 3 is on top, and 4 is on bottom. Here ${\text{Disc}}_{ij}^{cd}$ ${\text{Disc}}_{ij}^{cd}$ as equal to the distance amongst the midpoint of c on the direction D over Eij. For simplification of ${\text{Disc}}_{ij}^{cd}$ we took c as left eye distance as shown in Fig. 10. Here c (left eye distance) is the distance amongst the midpoint of left eye and around POIs. The ${\text{Disc}}_{ij}^{c2}$, ${\text{Disc}}_{ij}^{c3}$, and ${\text{Disc}}_{ij}^{c4}$ are the distances amongst the midpoint of left eye’s left-edge, right-edge, top-edge and, bottom-edge, respectively. Here c (right eye distance) is the distance amongst the midpoint of right eye and around POIs. The ${\text{Disc}}_{ij}^{c2}$, ${\text{Disc}}_{ij}^{c3}$ and ${\text{Disc}}_{ij}^{c4}$ are the distances amongst the midpoint of right eye’s left-edge, right-edge, top-edge, and bottom-edge, respectively. In case of mouth it is measured by ${\text{Disc}}_{ij}^{cd}$ d ∈ D and the distances amongst the midpoint of mouth and its 4 edges are measured by Eij. The bound_*c*_ is measured by Eq. (2).

(5) \begin{eqnarray*}{\text{bound}}_{c}=\min _{i,j,d}({\text{Disc}}_{ij}^{cd}),i\in I,j\in J,d\in D.\end{eqnarray*}

Let ${R}_{ij}^{c1},c\in C,1\in {L}_{c}$ be the region size which is equal to l. Here $Has{h}_{i}^{c1},c\in C,1\in {L}_{c}$ ${\text{Hash}}_{\mathrm{i}}^{\mathrm{cl}},\mathrm{c}\in \mathrm{C},\mathrm{l}\in {\mathrm{L}}_{\mathrm{c},}$ is the summation of distance amongst all c regions of subject i with a size of l. It is measured by Eq. (3).

(6) \begin{eqnarray*}{\text{Hash}}_{i}^{c1}=\sum _{x\not = y}\text{Algorithm}1({R}_{ix}^{c1},{R}_{iy}^{c1}).\end{eqnarray*}

Here x & y ∈ J. In order to match the c regions with i and size is l, we incorporated the following equation no 7.

(7) \begin{eqnarray*}{\text{Sim}}_{i}^{c1}= \frac{1}{Has{h}_{i}^{c1}+1} ,c\in C,1\in {L}_{c}.\end{eqnarray*}

Let Sim^*c*1^ be Sim^*cl*^ the average of ${\mathrm{Sim}}_{i}^{c1}$ among the 4 subjects. Where Sim^*c*1^ Sim^cl^ is the matching among c regions with size is equal to l. In lieu of individually c ∈ C, we took the size l to have maximum Sim^*c*1^in our experiment.

We have incorporated a searching method in detail is shown in Algorithm 2. During our experiment, we took 35 images from the CK + and 10 JAFFE databases, *i.e.*, Eij, i ∈I, j ∈J. To get proper output, the image sizes are kept the same (640×800) for all kinds of images.

**Algorithm 2: Searching for the OAROI**

Input: 45 (35 + 10) images selected from CK++ and JAFFE database

Output: the sizes of OAROI

a. for c in C
b. Compute bound_*c*_.
c. for i in I
d. for l in L
e. Compute $Has{h}_{i}^{c1}$
f. Compute ${\mathrm{Sim}}_{i}^{c1}$
g. Compute the MV of ${\mathrm{Sim}}_{i}^{c1}$ ${\mathrm{Sim}}_{\mathrm{i}}^{\mathrm{cl}}$ amongst 7 subjects and acquire Sim^*c*1^.
h. In drawing the picture of Sim^*c*1^, let l be start value of *X*-axis and Sim^*c*1^ be value of *Y*-axis.
i. Catch the size l from max value of Sim^*c*1^ andrecord the output.

**Figure 11:**
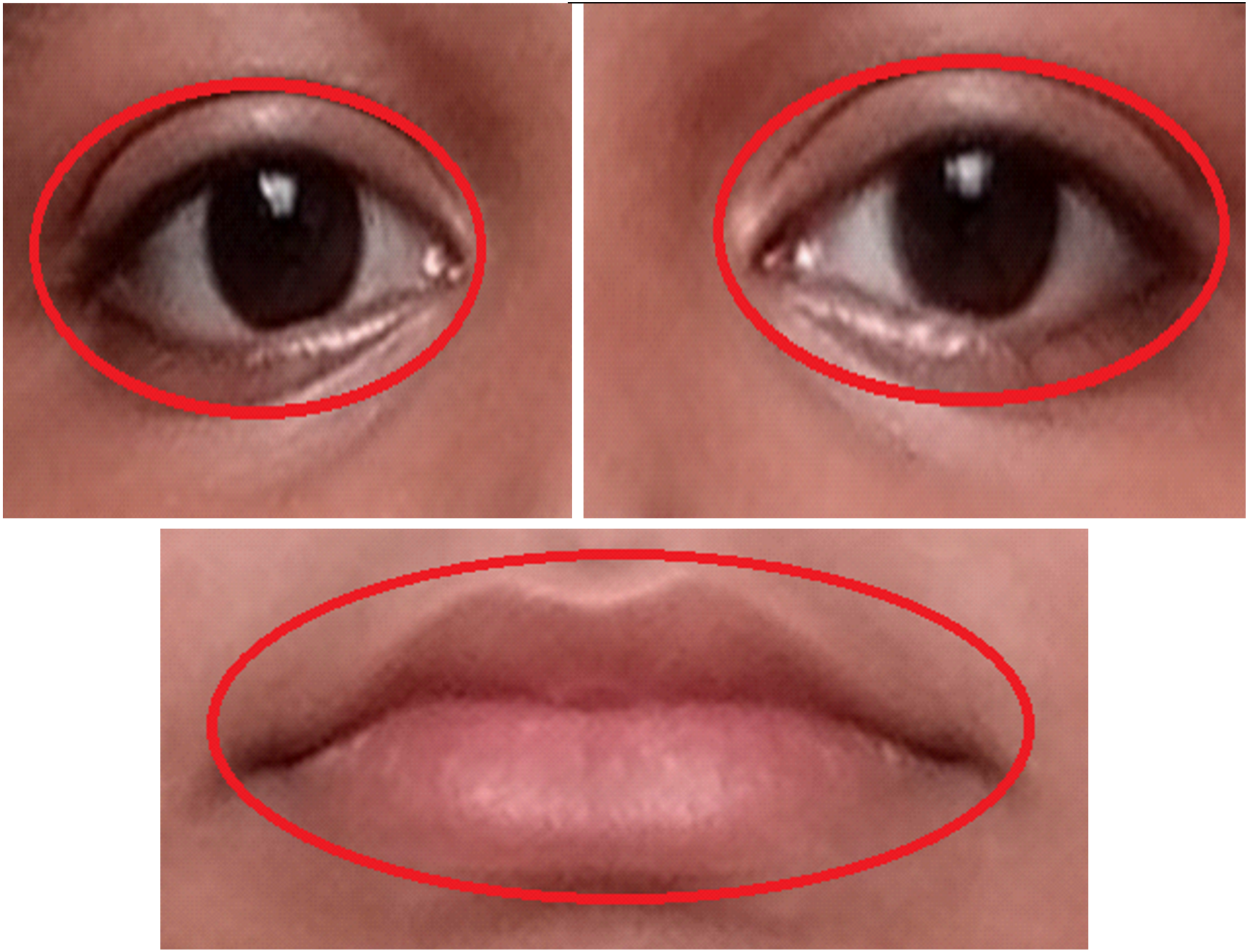
Examples of OAROI (left eye, right eye & lip).

Based on the Algorithm 2 the required picture will be following the step h. This is shown in Fig. 11. The sizes for the right-eye, left-eye, and lip regions are set to 160 × 120, 160 × 120, and 180 × 80 pixels. We found that the obtained size is half of the edge length of the OAROIs. As an outcome, the edge sizes of 3 kinds of OAROIs have been set to 140 × 80, 140 × 80, and 160 × 60 pixels, respectively. The OAROIs are shown in Fig. 9. It is found almost all active POIs are around the nose regions. For this reason, the nose region is kept aside of our experiment.

### Parallel face and facial AROI detection

One of the emergent objectives of this research is to speed up the process of facial expression with improved recognition accuracy. As stated earlier (shown in Fig. 1) the whole process is into three phases: Data processing, training, and testing of classified data. The data processing phase is executed in sequential order. In the first phase, images are recognized and then the following are carried out namely-pre-processing, POI detection, head-pose adjustment using rotation and ASHM method, image segmentation and AROI detection. Lastly, OAROI is identified based on our proposed searching method. The usage of OAROIs rather than the entire face helps us reduce data processing time and speed up processing of the first phase. The last two phases run in parallel since each ORAOI (namely the left-eye, right-eye, and lip region) is identified separately in parallel. This technique reduces the execution time for these two phases by 64% (approx) as shown in the experimental results. As per experimental calculation, only 1% of the total execution time is needed to complete the first phase and the last two phases took 99% (approx) of the total execution time. In applying parallelism, the system is three times faster. In calculating the required time in parallel processing we applied the famous formula of Amdahl’s Law (Gustafson, 1988) as mentioned below through Eq. (8).

(8) \begin{eqnarray*}\text{Speedup}= \left( \frac{1}{(1-P)+P/N} \right) .\end{eqnarray*}

Where P is the part that runs in parallel that is 0.99 (in our system), and N is the number of processors *i.e.*, 3. We use only three processors for simplification, as our system has 3 OAROIs and each one of them runs separately. Therefore, our parallel model is 2.94% faster, which is almost three times faster. We incorporated a Decision-Tree-Level Synthesis scheme that helps us to gain enhanced accuracy in recognition.

### Classification by decision-tree-level synthesis

**Figure 12:**
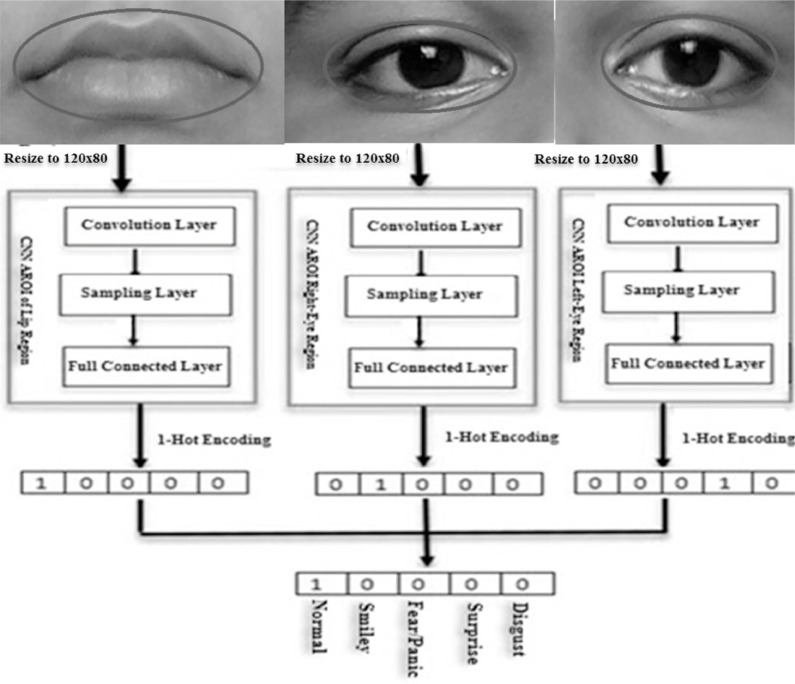
Schematic flow-diagram of learning features (feature-weight), expression classification and decision-tree-level synthesis method.

An emergent facial expression recognition technique demands a very high accuracy rate for recognition. In our study, the AROI-based CNN method is incorporated to extract features of the FER and their classification. Three OAROIs are chosen namely left-eye, right-eye, and lip regions. These three OAROIs are trained and classified in parallel using CNN. The final classification result is achieved which is remarkably higher by using the decision tree level synthesis technique. The schematic diagram (Fig. 12) depicts the applied technique.

The sizes of the optimized active regions (*i.e.*, the inputs of CNN) are dissimilar for each images. To make the sizes the same, we completed the following actions.

a. Optimized active regions of interest are resized to 120*80 pixels.
b. The CNN-based convolution layers and its connected sub-layers were applied to input images to extract features-weight and features.
c. Lastly, the execution is completed based on fully connected layer.

The feature weights have been applied to the fully-connected-layers to achieve the desired expressions. The One-hot (1) code is applied to detect desired expression. In our experiment we used five classes of expressions (Normal, Happiness, Fear, Surprise, and Disgust) in this study. So the length of one-hot (1) code is equal to five. Individually, one bit of one-hot code represents one expression. The index of 1 resultant from one expression is displayed. The bit zero (0) means False (No) and one (1) means True (Yes).

In the last phase, the final expression recognition result is achieved based on the decision-level synthesis method. This decision-level synthesis method is carried out based on the following:

\begin{eqnarray*}\text{Final Output}= \left\{ \begin{array}{@{}l@{}} \displaystyle j,if~2~\text{or more CNNs}\\ \displaystyle \text{classify the expression}\\ \displaystyle \text{as}~j~\forall ~j~\in ~J\\ \displaystyle \text{Here CNN result over left_}\text{eye, right_}\text{eye}~\mathrm{{\XMLAMP}}~\text{lip regions},\\ \displaystyle \text{otherwise}~0 \end{array} \right. \end{eqnarray*}

In order to classify any expression we need two or more classifiers as stated in the above expression. Here a class is indicated by j for all j ∈ J. The final outcome will be measured based on the j only. On the other hand, it is revealed that the desired classification is completed based on 3 CNNs. We considered 5 expressions (namely-Normal, Smiley, Fear, Surprise, and Disgust) in our experiment. If two classified expressions are “Normal” then the synthesis result will be “Normal”. If the results of the three CNNs are different then we have to choose the CNN result based on the highest accuracy. This has been demonstrated in the “Experimental Section”. Figure 12 shows the schematic flow diagram of the feature weight that we already calculated earlier and have shown mathematically through the decision-level-synthesis method as illustrated above.

## Experimental Evaluation

In these five expressions, five expressions are addressed. Based on the segmented images, an optimized AROI is introduced. The face areas/regions explored are the nose-tip, left-eye, right-eye, and lip regions. These four regions are the selected AROIs. The dimension of an active region is directly correlated with facial expression identification. An optimized searching algorithm is introduced to search for the appropriate dimension of active regions. An AROI of an appropriate size is called an OAROI. The eight divided segments are processed in parallel. A decision-tree-level-synthesis framework is built to classify FER. Among the 4 OAROIs, 3 (left-eye, right-eye, and lips) regions are chosen for our experiment. They are trained using CNN. The final classified expressions are achieved based on gained results. Our experiment is conducted based on the Extended Cohn-Kanade(CK+) (Kanade, Cohn & Tian, 2000; Lucey et al., 2010) and the JAFFE image sets which are freely available at http://web.mit.edu/emeyers/www/face_databases.html.

By applying parallelism the processing time has increased three times faster as compared to that of a sequential method. Since each region is processed individually, the results of these regions verify the accuracy of each other. This result confirms that the proposed technique achieves much-improved accuracy compared to earlier works (Salmam, Madani & Kissi, 2016; Mollahosseini, Chan & Mahoor, 2016; AlBdairi, Zhu & Mohammed, 2020; Almeida et al., 2020; Rajan et al., 2020).

During performance evaluation, we carried out both quantitative and qualitative performances. In our experiment, we used two datasets, CK+ and JAFFE, and their experimental results in expression recognition are shown in Tables 1 and 2.

**Table 1:** Performance detection accuracy of AROI with normal expression based on CK+ images.

**SL#**	**Images-set with normal** **expression**	**Nose-tip** **region (A) detection (%)**	**Left eye** **region (B) detection (%)**	**Right eye** **region (C)** **detection (%)**	**Lip region (D) detection (%)**
1	Image 72	91.1019109	91.2735824	92.46843956	91.19301281
2	Image 73	92.8138211	90.7120214	94.20602842	92.90663492
3	Image 74	93.6106171	93.7688752	95.01477636	93.70422772
4	Image 75	93.6454519	90.1541641	95.05013368	93.73909735
5	Image 76	89.2206315	89.0417528	90.55894097	89.30985213
6	Image 77	92.0991071	92.1111191	93.48059371	92.19120621
7	Image 78	91.1621711	91.1922011	92.52960367	91.25333327
8	Image 80	88.1097217	89.3309417	89.43136753	88.19783142
9	Image 81	92.9349421	91.8238321	94.32896623	93.02787704
10	Image 82	89.1865975	90.0020121	90.52439646	89.2757841
11	Image 85	90.6016111	90.3113211	91.96063527	90.69221271
12	Image 87	93.2652721	93.1934003	94.66425118	93.35853737
13	Image 88	91.2229318	90.4114211	92.59127578	91.31415473
14	Image 91	92.7427501	93.3761829	94.13389135	92.83549285
15	Image 92	89.8216319	88.2253372	91.16895638	89.91145353
16	Image 95	92.4109186	90.2529628	93.79708238	92.50332952
17	Image 96	92.2938016	91.2046135	93.67820862	92.3860954
18	Image 100	91.3924011	90.3498596	92.76328712	91.4837935
19	Image 102	93.2948016	91.2046135	94.69422362	93.3880964
20	Image 106	91.0923013	90.3655753	92.45868582	91.1833936
21	Image 107	90.2613712	89.5112218	91.61529177	90.35163257
22	Image 109	91.3004092	90.2636735	92.66991534	91.39170961
23	Image 110	93.2696765	93.9670732	94.66872165	93.36294618
24	Image 111	90.2222321	90.8094187	91.57556558	90.31245433
25	Image 113	90.1131231	90.2914012	91.46481995	90.20323622
26	Image 114	93.2696765	92.4050127	94.66872165	93.36294618
27	Image 115	92.1046126	92.1932011	93.48618179	92.19671721
28	Image 117	92.4140217	91.3894982	93.80023203	92.50643572
29	Image 119	91.9839921	89.1932041	93.36375198	92.07597609
30	Image 122	92.9339411	90.2931029	94.32795022	93.02687504
31	Image 127	93.1661731	90.3643741	94.5636657	93.25933927
32	Image 128	92.8139212	89.2905013	94.20613002	92.90673512
33	Image 130	91.2766855	92.5040116	92.64583578	91.36796219
34	Image 132	90.3653751	88.9993104	91.72085573	90.45574048
35	Image 138	93.2389458	92.2336415	94.63752999	93.33218475
	**Average**	**91.73593**	**90.91469816**	**93.11196895**	**91.82766593**

**Table 2:** Performance detection accuracy of AROI with normal expression based on JAFFE images.

**SL#**	**Images-set with normal** **expression**	**Nose-tip (A) detection (%)**	**Left eye (B) detection (%)**	**Right eye(C)** **detection (%)**	**Lip (D) detection (%)**
1	Image 1	86.490246	86.70647162	89.21468875	90.413856
2	Image 2	88.097834	88.31807859	90.87291577	90.81164
3	Image 3	88.846074	89.06818919	91.64472533	90.345665
4	Image 4	89.818786	90.04333297	92.64807776	90.3528835
5	Image 5	83.78361	83.99306903	86.42279372	84.7715694
6	Image 6	88.366674	88.58759069	91.15022423	89.9210442
7	Image 7	87.486834	87.70555109	90.24266927	85.457184
8	Image 8	82.740398	82.947249	85.34672054	86.767776
9	Image 9	89.151574	89.37445294	91.95984858	89.173006
10	Image 10	84.69165	84.90337913	87.35943698	89.17004
	**Average**	**86.947368**	**87.16473642**	**89.68621009**	**88.71846641**

In the experiment, the nose-tip region is the central point of the AROI. So, the detected value for a normal expression was kept fixed for the corresponding image set for all types of expression (Normal, Happiness, Fear, Surprise, and Disgust).

### Used databases

The CK+ *i.e.*, Extended Cohn-Kanade database contains 327 male and female images, and each of them has seven types of expression. Out of them, we use five (*i.e.*, Normal, Happiness, Fear, Surprise, and Disgust) types in our experiment. To get the proper expression as a sample image, we select one from the last four frames in this experiment. The JAFFE image database comprises 213 facial expressions from 10 females and each of them has seven types of expression. However, we select five, *i.e.*, Normal, Happiness, Fear, Surprise, and Disgust. In the JAFFE database, it is found that for each expression (subject), there are three images. The chosen images are kept in the database. We used all images from the JAFFE database in our experiments.

In this study, we took the images detectable naturally for our experimental work. In deep learning techniques, huge training data sets are needed to achieve better performance. The total volume of available data from both databases was not enough for CNN based training under the deep learning method. To yield more data, firstly the original image is flipped to horizontal. Secondly, histogram equalization is conducted on both flipped and original images. Lastly, they have been converted to gray and the pose estimation algorithm (PEA) is also applied. By applying the above scheme the total volume of data has increased by 8 times. Moreover, we applied the segmentation technique that has divided each image into four classes. In this way, the total volume of images became 32 times that of the original one. The OAROIs of all the images are saved in the info-image-data-mask database for training and testing. In addition, we also took the image sets separately as samples of two databases and trained them to classify them based on by Happy & Routray (2015) method. Our experimental result and the time of execution are better as compared to other researchers.

Tables 1 and 2 illustrate the performance of execution for normal expressions of two databases CK+ and JAFFE. Table 3 shows the average execution accuracy of 5 types of expression based on CK+ images. The last row of each table shows the average accuracy of each OAROI. Table 3 illustrates the performance of recognition for a selected image set of 35 images taken from the CK+ image database and their execution time in milliseconds in the sequential method. Table 4 demonstrates performance in recognition based on the CK+ image measured in millisecond. Table 5 shows the performance of recognition and their execution time in milliseconds by applying parallelism. The accuracy exceeds 91% in all cases and the average accuracy of the whole classification process reaches 96.23%.

**Table 3:** Average performance accuracy in detection (%) of facial expression of AROI of region (A, B, C & D) based on CK+ images.

**Facial expression of CK+**	**Nose-tip** **region (A) detection (%)**	**Left eye region (B) detection (%)**	**Right eye region (C) detection (%)**	**LIP** **region (D) detection (%)**	**Proposed OAROI detection (%)**
**Normal**	91.73593	90.91469816	93.11196895	91.82766593	**95.43976817**
**Smiley**	91.73593	89.44253175	90.35989105	94.94668755	**97.32035474**
**Panic**	91.73593	92.10287372	92.46981744	93.21196895	**96.10153999**
**Surprise**	91.73593	92.19460965	93.11196895	95.86404685	**98.26064802**
**Disgust**	91.73593	89.99294733	90.91030663	91.36898628	**94.02932825**

**Table 4:** Performance matrix of selected CK+ image set based on execution time (ET) in milliseconds (MS).

	**Normal face images ET in MS in SM**			**Smiley face images ET in MS in SM**			**Panic face images ET in MS in SM**			**Surprise face images ET in MS in SM**			**Disgust face images ET in MS in SM**		
**Image** **sets**	Right eye ET	Left eye ET	Lip ET	Right eye ET	Left eye ET	Lip ET	Right eye ET	Left eye ET	Lip ET	Right eye ET	Left eye ET	Lip ET	Right eye ET	Left eye ET	Lip ET
**Total ET**	390.647	384.45	421.996	386.74	380.6	451.54	396.45	390.23	426.94	402.37	395.982	451.54	351.58226	346.00329	400.8966
**Average ET**	11.1613	10.984	12.057	11.05	10.87	12.901	11.327	11.149	12.198	11.496	11.3138	12.901	10.045207	9.8858083	11.45419

**Table 5:** Comparing the Execution Time (ET) in milliseconds of parallel method and sequential method for CK+ image set.

Parallel ET for normal face images	12.05	Sequential ET for normal face Images	34.2
Parallel ET for smiley face images	12.9	Sequential ET for smiley face images	34.8
Parallel ET for panic face images	12.19	Sequential ET for panic face images	34.67
Parallel ET for surprise face images	12.9	Sequential ET for surprise face images	34.7
Parallel ET for disgust face images	11.45	Sequential ET for disgust face images	34.38
Average ET for parallel Method	12.299	Average ET for sequential method	34.158

Table 6 illustrates the average execution accuracy of Normal, Happiness, Fear, Surprise, and Disgust for JAFFE images and the accuracy of the proposed synthesis method (our model) in the process of OAROI detection. The last row of each table shows the average accuracy of each OAROI. The accuracy exceeds 88% in all cases and the average accuracy of the whole classification process reaches 96.23%.

**Table 6:** Shows the execution time (ET) in milliseconds for all OAROI using JAFFE images considering the five targeted expressions.

**Facial expression of JAFFE**	**Nose-tip** **region (A) detection (%)**	**Left eye region (B) detection (%)**	**Right eye region (C) detection (%)**	**LIP region (D) detection(%)**	**Proposed OAROI detection (%)**
**Normal**	86.947368	87.16473642	89.68621009	88.71846641	91.92836534
**Smiley**	86.947368	89.33842062	90.03399956	91.16431535	93.44342323
**Panic**	86.947368	88.34722062	88.79934694	88.2950522	91.01933061
**Surprise**	86.947368	90.2078943	91.04258903	92.1207364	94.42375481
**Disgust**	86.947368	85.29536801	86.51263116	87.07778905	89.25473378

Table 7 illustrates the performance of recognition for the selected image set of 10 images taken from the JAFFE image database and their execution time in milliseconds in the sequential method has been shown. In Table 8, with the same images, the performance of recognition and their execution time in milliseconds in the parallel method has been shown.

**Table 7:** Performance matrix of selected JAFFE image set based on execution time (ET).

	**Normal face images ET in MS in SM**			**Smiley face images ET in MS in SM**			**Panic face images ET in MS in SM**			**Surprise face images ET in MS in SM**			**Disgust face images ET in MS in SM**		
**Image sets**	Right eye ET	Left eye ET	Lip ET	Right eye ET	Left eye ET	Lip ET	Right eye ET	Left eye ET	Lip ET	Right eye ET	Left eye ET	Lip ET	Right eye ET	Left eye ET	Lip ET
**Total ET**	103.8401	102.9574	115.4043	102.8017	101.92783	123.4826	104.8785	103.98697	116.55834	106.9553	106.04612	123.4826	93.45609	92.66166	109.63409
**Average ET**	10.38401	10.29574	11.54043	10.28017	10.192783	12.34826	10.48785	10.398697	11.655834	10.69553	10.604612	12.34826	9.345609	9.266166	10.963409

**Table 8:** Comparing the Execution Time (ET) in milliseconds of parallel method and sequential method for JAFFE image set.

Parallel ET for normal face images	11.54	Sequential ET for normal face images	32.22
Parallel ET for smiley face images	12.34	Sequential ET for smiley face images	32.82
Parallel ET for panic face images	11.65	Sequential ET for panic face images	32.54
Parallel ET for surprise face images	12.34	Sequential ET for surprise face images	33.64
Parallel ET for disgust face images	10.96	Sequential ET for disgust face images	29.57
Average ET for parallel method	11.75	Average ET for sequential method	32.16

Moreover, Table 8 compares the ET in milliseconds for our proposed synthesis method (parallel execution) with the sequential one for the JAFFE image set. It shows the ET for our proposed parallel method reduces to 36.536% than that of a sequential method.

### Used CNN structure and tenfold validation

**Figure 13:**
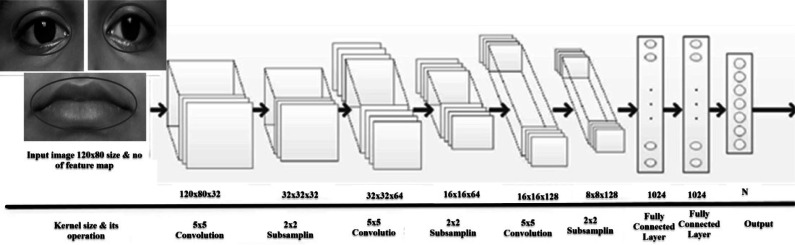
The structure of proposed CNN with three convolution, three sub-sampling layers and two fully connected layers.

The proposed CNN structure is shown in Fig. 13. In Fig. 12 it is shown that the system receives gray images as input with a fixed size of 120*80 pixels. Three CNN layers are offered along with AROIs. Here for each of them is interlinked via a sub-sampling layer. A relationship is built up by kernel sizes in CNN layers. The measurement of sub-sampling layers are 5 × 5 and 2 × 2, correspondingly. Every sub-sampling layer should be multiple of 2. In Fig. 13, it is being seen that the size after 1st convolution became 120*80*32. A similar method is applied to the rest of the following layers. There are 1,024 neurons in total. Each of two are fully connected. In this way, CNN helps us to gain better results. The performance is achieved based on the voting score of five dissimilar classified expressions. The output (shown in Fig. 13) is determined based on the number of dissimilar images. The proposed CNN structure (as shown in Fig. 13) illustrates how convolution works with the sub-sampling layer and fully connected layer.

### Experiments on ten-fold cross-validation

**Figure 14:**
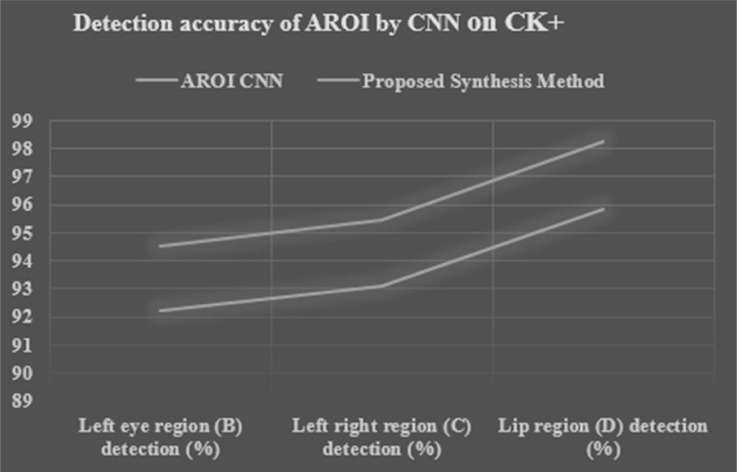
Performance detection accuracy chart of each AROI by CNN and proposed synthesis method result in tenfold cross validation based on CK+ image set.

**Figure 15:**
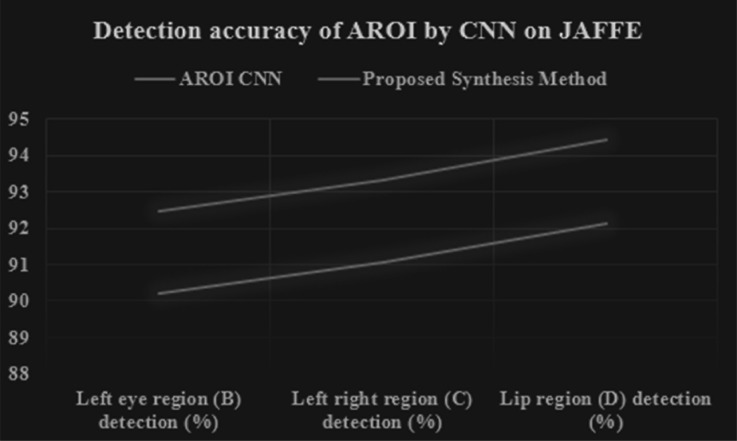
Performance detection accuracy chart of each AROI by CNN and proposed synthesis method result in tenfold cross validation based on JAFFE image set.

We applied ten-fold cross-validation to calculate the performance of the proposed synthesis method and it is compared with its original CNN. Tables 9 and 10, Figs. 14 and 15 show the performance accuracy of each AROI. This performance is achieved based the proposed ten-fold cross-validation that we applied on two image datasets. Here we applied our proposed synthesis method. By using the CK+ image set the performance based on three CNNs (on left-eye, right-eye, and lip region) are 92.19%, 93.11%, and 95.86% respectively, whereas by using the JAFFE image set the performances are gained 90.207%, 91.042%, and 92.120% respectively. Our achievement is excellent as compared to other researchers (Zhang & Wang, 2020; Hossain & Sanyal, 2012b; Moghaddam, Akbarizadeh & Kaabi, 2019; Cheon & Kim, 2008; AlBdairi, Zhu & Mohammed, 2020). Moreover, our proposed synthesis method gained an excellent rate of higher accuracy, *i.e.*, 94.499%, 95.439%, and 98.26% respectively in the CK+ set and 92.463%, 93.318%, and 94.423% respectively in the JAFFE image set. In contrast, the researchers (Happy & Routray, 2015) have conducted their experiments based on fused data from the CK+ and JAFFE databases, and their accuracies were 89.64% and 85.06% respectively, which are remarkably less than that of our results. Liu et al. (2016) achieved accuracies of 92.40% and 63.40% respectively. Zhong et al. (2012) used CSPL method and they used MNI and CK+ dataset with the accuracy of 73.53% and 89.89% respectively. Lee, Plataniotis & Ro (2014) applied SRC in their dataset MNI,JAFFE and CK+ and they achieved 93.81%,87.85% and 94.70% respectively. Mollahosseini, Chan & Mahoor (2016) achieved accuracies of 93.20% and 77.90% respectively. In their experimentation, they tried to locate active patches around the nose, mouth, and eye regions. Our achieved result is a robust one as compared to other researchers. Moreover, we can gain 2.8% higher accuracy by introducing a decision-level synthesis method. This confirms the effectiveness and robustness of our proposed method.

**Table 9:** Performance detection accuracy of AROI by CNN and proposed synthesis method based on CK+ image set.

**Method applied**	**Left eye region (B) detection (%)**	**Right eye region (C) detection (%)**	**LIP region (D) detection (%)**
**AROI CNN**	92.19460965	93.11196895	95.86404685
**Proposed synthesis method**	94.49947489	95.43976817	98.26064802

**Table 10:** Performance detection accuracy of AROI by CNN & proposed synthesis method based on JAFFE image set.

**Method applied**	**Left eye region (B) detection (%)**	**Right eye region (C) detection (%)**	**LIP region (D) detection (%)**
**AROI CNN**	90.2078943	91.04258903	92.1207364
**Proposed synthesis method**	92.46309166	93.31865376	94.42375481

### Discussion on the experimental results

The experiment was completed using OS-Window 7, Language- Python 3.6, Intel Core (TM) i7 with a CPU processing speed of 3.40 GHz with RAM size is 6 GB. It is a Laptop with 4 physical cores. The maximum amount of experimental time is spent for dataset training. We know that training a dataset in a deep learning method needs a longer time as compared to other techniques. Because training time is correlated with several factors, like the volume of the data, the model of a structure, calculating the power, and so on.

As per the information shown in Table 11, it is clear that the CK+ database takes more time than the JAFFE database because there are more images in the CK+ database with complex conditions. In addition, the same reason is applicable for the training time for the Info-image-data-mask database as compared with the independent databases. Indeed, the Info-image-data-mask database includes both images of two databases. Moreover, ten-fold cross-validation is also applied in our experiment to increase the volume of data sets. As a result of which we have acquired a huge volume of data which is necessary for deep learning methods for better result.

**Table 11:** Average training time required for two databases and the info-image-data-mask.

**Database**	**Required time**
CK+	8 min 22 s
JAFFE	2 min 3 s
Info-image-data-mask	40 min 7 s

We did not use other dataset like the NVIE (Nada & Heyam, 2020; Cheon & Kim, 2008; Wang & He, 2013; Shen, Wang & Liu, 2013; Rajan et al., 2020) or fused database because of two reasons. Firstly, most of the NVIE image database contains thermal images. Secondly, it is not very reliable as it is found from the performance analysis results of researchers (Cheon & Kim, 2008; Wang & He, 2013; Shen, Wang & Liu, 2013) that results gained from NVIE dataset differ depending on the method used. Their result analysis have been shown with the Table 12. The Table 12 illustrates that the achieved results varies depending on applied methods based on same database *i.e.*, NVIE. Whereas the JAFFE and CK+ are very authentic and filtered. So we have chosen these two image sets in our application. Moreover, we concentrated on the required time for execution, so, for these two reasons, we applied the CK+ and JAFFE datasets.

**Table 12:** Performance result analysis of different researchers by applying different method based on NVIE database.

**Researchers**	**Data set**	**Used method**	**Overall accuracy**
Cheon & Kim (2008)	NVIE	AAM (Active appearance model)	66.24%
		integrated + AAM	71.72%
		DAF	79.16%
		integrated + DAF	91.52%
Wang & He (2013)	USTC-NVIE	AAM + Thermal statistical parameters KNN	63%
		Bayesian networks (BNs)	85.51%
Shen, Wang & Liu (2013)	USTC-NVIE	KNN	73%

Due to a diverse experiment setting, it is not easy to make an impartial evaluation of the proficiency of dissimilar methods. AlBdairi, Zhu & Mohammed (2020) have proposed that their CNN-based deep learning method has achieved 96.9% accuracy in the verification of the human face. Rajan et al. (2020) have proposed their CNN and LSTM-based methods where they have used MMI, SFEW, and their dataset. They achieved the accuracy of 81.60%, 56.68%, and 95.21% respectively. Hossain, Samanta & Sanyal (2012c) trained their data for CNN and it took about 20 min. They used the CK+ database in their experiment. Their hardware configuration was an Intel Core i7 CPU of 3.4 GHz. Liu et al. (2016) trained their data by using eight-fold cross-validation. Their hardware configuration was 6 core with a processing speed of 2.4 GHz. Their experiment took about 8 days to complete. The comparison of performance and its result analysis with different researchers are shown in a tabular form. It is shown by the following Table 13.

**Table 13:** Performance result analysis of different researchers.

**Researchers**	**Method used**	**Accuracy based on used datasets**				
		JAFFE	CK+	MNI	SFEW	OWN
Happy & Routray (2015)	SFP	85.06%	89.64%			
Liu et al. (2016)	AUDN, Eight-fold cross-validation	63.40%	92.40%			
Zhong et al. (2012)	CSPL		89.89%	73.53%		
Lee, Plataniotis & Ro (2014)	SRC	87.85%	94.70%	93.81%		
Kanade, Cohn & Tian (2000)	DNN		93.20%	77.90%		
Shen, Wang & Liu (2013)	CNN and LSTM-based methods ten-fold cross-validation			81.60%	56.68%	95.21%
Our applied method	CNN by applying on 1. left-eye 2. right-eye 3. lip region	90.207% 91.042% 92.120%				
Our proposed method	Decision-level synthesis method and ten-fold cross-validation by applying on 1. left-eye 2. right-eye 3. lip region		94.499% 95.439% 98.260%			
Our Achievement	**We gain 2.8% higher accuracy by introducing a decision- level synthesis method and ten-fold cross-validation**					

## Conclusions

In our investigation, a sophisticated and proficient facial expression recognition scheme is introduced for classifying five different types of expressions. In our experiment, we considered only four active regions namely nose-tip, right-eye, left-eye, and lip regions. A search method called the Optimized active Region of Interest (OAROI) is introduced for faster searching of active regions. Among the four AROIs, three regions (right eye, left eye, and lip) are chosen for experimentation. The required data sets are trained by using AROI-based CNN to classify expressions. A pose estimation algorithm is also introduced. An approach “rotate and correct” based on the exact pose is also incorporated. A decision-level synthesis method is applied to gain higher recognition accuracy. Experimentation on both separate databases and info-image-data-mask databases is voted for to assess the performance of the proposed technique.

To increase the volume of the image set for better recognition accuracy, we proposed a ten-fold-cross validation method where CNN is used. By using the CK+ image set we achieve an accuracy of 92.19%, 93.11%, and 95.86% respectively for the left-eye, right-eye, and lip region, whereas by using the JAFFE dataset we achieve the accuracies of 90.207%, 91.042%, and 92.120% respectively. This achievement is excellent as compared to other researchers (Happy & Routray, 2015; Liu et al., 2016; Mollahosseini, Chan & Mahoor, 2016; AlBdairi, Zhu & Mohammed, 2020; Rajan et al., 2020). Moreover, we introduce a parallel processing technique that has accelerated the system’s performance to a greater extend. The AROIs are processed in parallel, so processing time is almost three times faster than that of a sequential method. By introducing our proposed method we achieved 95.27% in recognition. Our proposed synthesis method helped us to gain excellent accuracy, *i.e.*, 94.499%, 95.439%, and 98.26% respectively in CK+ and 92.463%, 93.318%, and 94.423% respectively in the JAFFE image set. However, we gain 2.8% higher accuracy by introducing a decision-level synthesis method. This confirms the effectiveness and robustness of the synthesis method. In the future, a few other datasets will be used to check our results. In that work, AI will be incorporated along with more than ten-fold cross-validation.

## Supplemental Information

10.7717/peerj-cs.894/supp-1 Supplemental Information 1 Program Code

## Additional Information and Declarations

### Data Availability

The following information was supplied regarding data availability: The code are available in the Supplemental Files. The CK+ Image Set is available at Access to the JAFFE Images can be requested at: https://zenodo.org/record/3451524/accessrequest.
